# Single Camera-Based Gait Analysis Using Pose Estimation for Ankle-Foot Orthosis Stiffness Adjustment on Individuals With Stroke

**DOI:** 10.1109/JTEHM.2025.3585442

**Published:** 2025-07-02

**Authors:** Masataka Yamamoto, Koji Shimatani, Daisuke Matsuura, Yusuke Murakami, Naoya Oeda, Hiroshi Takemura

**Affiliations:** Department of Mechanical and Aerospace EngineeringTokyo University of Science 26413 Noda Chiba 278-8510 Japan; Course of Physical TherapyPrefectural University of Hiroshima 12798 Mihara Hiroshima 723-0053 Japan; Department of Rehabilitation MedicineSchool of MedicineFujita Health University 12695 Toyoake Aichi 470-1192 Japan; Department of RehabilitationBrain Attack Center, Ota Memorial Hospital Fukuyama Hiroshima 720-0825 Japan

**Keywords:** Ankle-foot orthosis, gait analysis, pose estimation

## Abstract

Introduction: Stroke is one of the most common causes of impaired gait. The use of an ankle-foot orthosis (AFO) is one of the recommended methods to improve gait function in stroke patients. Although the stiffness of the AFO is adjusted for each stroke patient, the effect of stiffness adjustment remains unclear due to the difficulty in measuring the gait parameters in a clinical setting. Objective: This study aimed to investigate the effect of adjusting the AFO stiffness based on the gait ability of stroke patients using a markerless gait analysis method. Methods: A total of 32 individuals with stroke were directed to walk under five conditions: no-AFO and AFO with four different levels of spring stiffness. These springs were used to resist the plantarflexion movements. Moreover, the best gait speed improvement condition (best condition) was determined from the five gait conditions for each participant and was compared with the other conditions, assuming a clinical setting. Spatiotemporal gait parameters such as the gait speed, cadence, step length, stance phase, and swing phase were measured from body keypoints in RGB images. Results and Conclusion: The experimental results showed that the gait speed, cadence, step length on both sides, and stance time on both sides were significantly improved in the best condition compared with the other conditions. This study demonstrated the usefulness of the markerless gait analysis method using a single RGB camera and the effectiveness of AFO stiffness adjustment based on the gait ability of the users.

Clinical Impact Statement: This study can contribute towards enhancing the therapeutic effect of gait rehabilitation with AFO and for improving quality of life by recovering the gait function.

## I. Introduction

Recovering the gait function is an important objective in individuals with stroke. Post-stroke gait is commonly limited in symmetrical gait patterns, gait speed, and step length owing to various impairments such as hemiplegia and sensory disorders [1], [2]. Ankle-foot orthoses (AFO) are widely used to improve the post-stroke gait function. The use of an AFO on the hemiparetic side of the lower limb can improve the gait speed, energy expenditure, and balance [3], [4], [5].

Among the various parameters of an AFO, stiffness is one of the most important for adjusting the body function of each individual. In particular, the stiffness of the plantarflexion resistance function of the AFO is effective for the gait of individuals with stroke because it is difficult for them to maintain the dorsiflexion position during the swing phase and perform a smooth weight transfer to the hemiparetic side during the stance phase. One of the key functions of this AFO is to assist the eccentric contraction of the ankle dorsiflexors muscle during the loading response of the hemiparetic side, which can resist rapid ankle plantarflexion from heel contact to foot flat. Individuals with reduced this function by stroke may be improved gait function with the use of AFO. Plantarflexion resistance function can assist in the ankle dorsiflexion torque, prevent toe drag, affect the joint moment, and improve the weight acceptance response [6], [7], [8]. Previous studies have reported that the magnitude of AFO stiffness affects the spatiotemporal gait parameters, such as gait speed and step length, as well as joint kinematics and kinetics [6], [9], [10]. In contrast, another study reported that AFO stiffness did not affect gait speed or joint kinetics [11]. These contradictory reports may be because of the simple comparison of various stiffness AFO conditions in stroke patients with different symptoms and gait performance. In a clinical setting, the magnitude of this stiffness is adjusted for each individual with stroke based on the gait performance and body function. To the best of our knowledge, no previous research has investigated the effectiveness of adjusting AFO stiffness based on individual gait abilities, similar to adjustments of AFO made in clinical setting. Therefore, the effect of AFO stiffness adjustment for individuals with stroke should be verified by comparing good adjustment conditions for the gait performance of each user with other stiffness conditions.

To evaluate the gait performance, a marker-based optoelectronic motion capture system is the gold standard method owing to its high reliability and repeatability [12], [13]. However, it is difficult to use this method in clinical settings because it is time-consuming and requires special environments. Walking several times under multiple conditions, such as AFO stiffness adjustments for each individual, is not pragmatic. Recently, markerless motion capture methods have been proposed as alternatives to marker-based optoelectronic gait analysis systems. These methods often use other sensors such as cameras, pressure sensors, and inertial measurement units [14], [15].

Markerless motion analysis using a depth camera or a single RGB camera has the potential to be used in clinical settings because it does not require specialized environments, high measurement time, or considerable cost [16], [17]. Human pose estimation methods using RGB images or videos, such as Openpose [18] can be used for two-dimensional markerless gait analysis. Despite the limitations in the accuracy of joint angles, markerless gait analysis based on a single RGB camera using Openpose can accurately measure spatiotemporal gait parameters and a few of the joint kinematics under various gait conditions [19]. These RGB camera-based motion analysis systems were verified as clinical screening tools for Parkinson’s disease and autism spectrum disorders [20], [21]. Therefore, a single RGB camera-based gait analysis method can verify the effect of AFO stiffness adjustment for stroke patients, while enabling ease of use in clinical settings. If we can easily determine the effect of adjusting the AFO stiffness for each individual with stroke, it can increase the effectiveness of stroke gait rehabilitation and provide useful information for therapists and patients. This study aimed to investigate the effect of AFO stiffness adjustments based on the gait ability of each individual with stroke using a markerless gait analysis method.

## II. Methods

### A. Participants and AFO Conditions

A total of 32 inpatients with stroke participated in this study. All participants received conventional rehabilitation for seven days a week during their hospitalization. The inclusion criteria were unilateral ischemic or hemorrhagic stroke, first-time stroke, at least 30 days after the onset of stroke, and the ability to walk 20 meters independently with or without supervision. The exclusion criteria were age <20 years, inability to follow verbal requests, history of an orthopedic surgery in the year before participating in this study, trunk or leg pain during walking and standing, and severe comprehensive aphasia or unilateral spatial neglect. All the procedures were approved by the Ethics Committee of the Brain Attack Center, Ota Memorial Hospital (162). Informed written consent was obtained from all the participants prior to the experiment. Table 1 presents the characteristics of the study participants. Owing to the inclusion criteria, low scores on the functional ambulation category (category 0, 1, 2), an indicator for evaluating the gait ability, and low scores on the lower limb Brunnstrom stage (stage 1, 2), an indicator for evaluating the motor function of individuals with stroke, were not included. This study also investigates changes in gait function with and without AFO, participants who did not use an AFO during gait training on the day of measurement were included. The most of participants used AFO during gait training, and all of them used an articulated type AFO.

**TABLE 1:** Characteristics of the Participants

Characteristics	Values
Sex (male/females)	(18/14)
Age (years)	$66.4 \pm 9.4$
Height (m)	$1.57 \pm 0.08$
Weight (kg)	$55.5 \pm 8. \beta $
Type of stroke (ischemic/hemorrhagic)	(28/4)
Hemiparetic side (left/right)	(19/13)
Time since to stroke (days)	$74.6 \pm 36.9$
Functional Ambulation Category (3/4/5)	(7/15/10)
Lower limb Brunnstrom Stage (3/4/5)	(5/9/18)
Use of AFO during gait rehabilitation (Yes/No)	(22/10)

Values represented mean ± standard deviation or number of participants (n).

**FIGURE 1.**
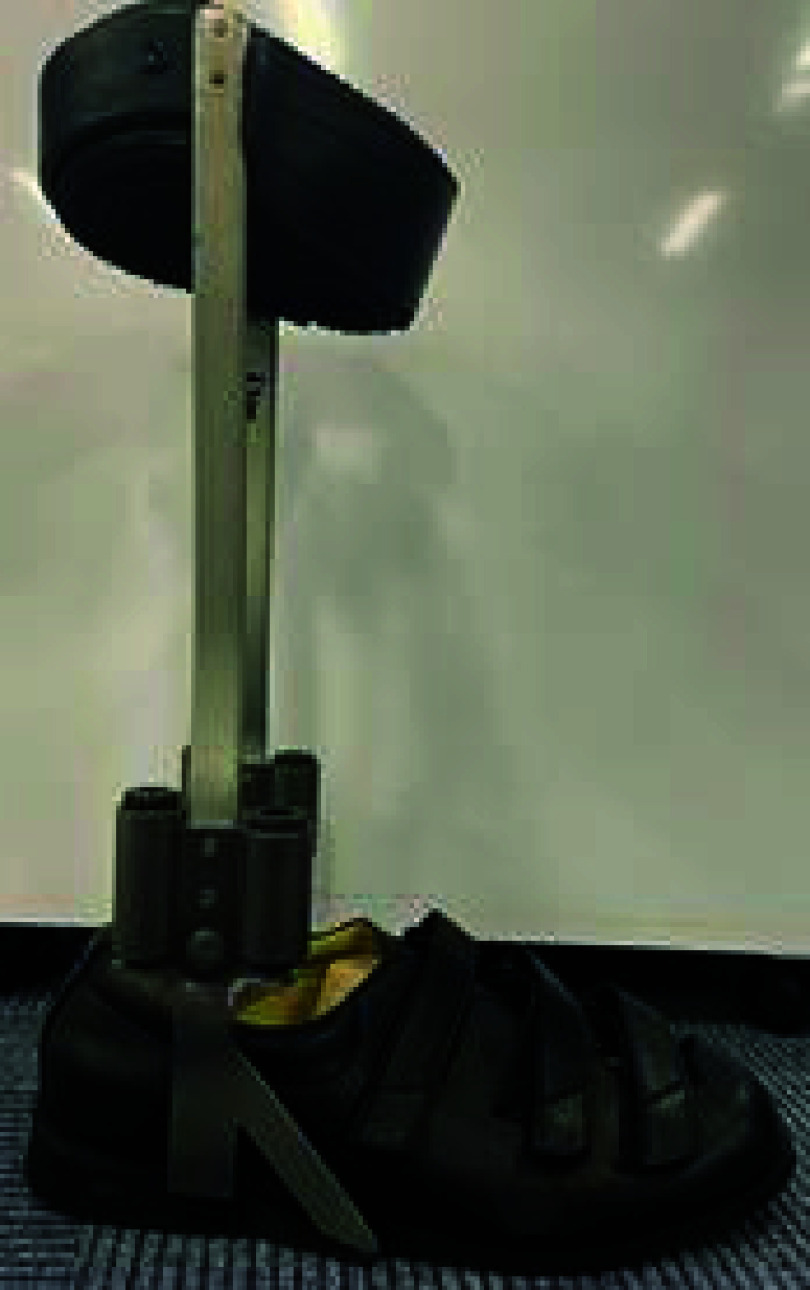
An articulated stiffness adjustable AFO.

An articulated AFO was used in this study (Fig. 1). This AFO generates a plantarflexion resistive moment by inserting silicon chromium steel springs into the ankle articulations [22]. The AFO comprised a cuff, strut, metal ankle articulation, and shoe. The total weight of the AFO is 1.34 kg. In this study, four springs (1–4) were selected for the AFO. The magnitude of the AFO stiffness conditions by the springs were as follows: spring 1, 0.64 Nm/deg; spring 2, 1.14 Nm/deg; spring 3, 1.57 Nm/deg; and spring 4, 1.74 Nm/deg. If these springs are not used, the AFO can perform ankle dorsiflexion and plantarflexion without a resistive moment.

Power analysis was used to determine the required sample size (alpha level, 0.05; power, 0.80; effect size, 0.25), which was 26.

### B. Experimental Setting and Procedure

**FIGURE 2.**
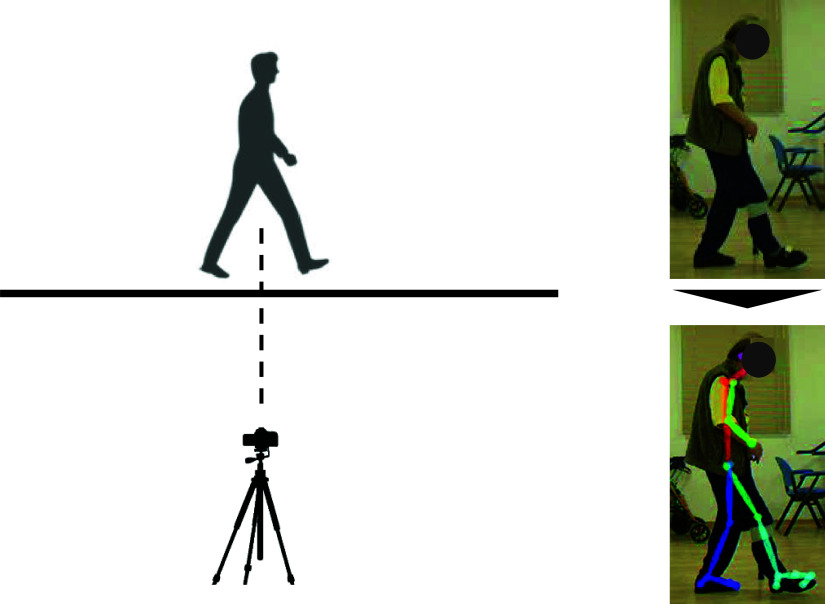
Overview of the experimental set-up and key-points estimation from the RGB image. Left side: participants walked on a 15 m straight walk way, and an RGB camera was placed at 4 m away from the walk way for capturing motion in the sagittal plane. Right side: body keypoints obtained from the RBG images were used for calculating the temporospatial gait parameters.

To measure the spatiotemporal gait parameters, a single RGB camera (Basler AG, Ahrensburg, Germany) was placed on the hemiparetic side of each participant to capture the motion of the sagittal plane. The pixel resolution and sampling rate were $720\times 520$ and 100 Hz, respectively. Single RGB camera was chosen for the measurement in this study because it is usually used in a clinical setting. For the calculation of spatiotemporal parameters such as step length, we converted pixels to meters using a 0.1-meter tape. Additionally, we used the sampling rate for time-related parameter calculations. Only one RGB camera was used for ease of use in clinical settings. Therefore, the preparation of the setup was very simple, and the camera was connected to a laptop using a wire. The camera was placed 4 m away from the straight walkway and its height was set at the greater trochanter of each participant. The spatiotemporal gait parameters were calculated from the body key-points using OpenPose. OpenPose is an open-source human pose estimation system that estimates the human joint location and feature points from each RGB image using a two-branched multistage convolution neural network [18]. First, the part-affinity fields, which form a set of 2D vector fields for the location and orientation of the body segments in the images, were predicted. The confidence maps of the locations of each segment were then predicted. Finally, both the stages were analyzed to output the 2D body key-points for each image. Further details on OpenPose can be found in the study by Cao et al. [18]. The Body _25 OpenPose model was used in the study. This model consists of 25 body keypoints: the nose, neck, mid-hip, and bilateral key-points of the eyes, ears, shoulders, elbows, wrists, hips, knees, ankles, heels, big toes, and small toes. OpenPose estimates 25 two-dimensional body keypoints in RGB images during walking (Fig. 2).

The participants were asked to walk under five conditions: no-AFO and AFO with springs 1–4. Under the spring 1–4 conditions, the participants wore the AFO on their hemiparetic lower limb and the same type of shoe on the other side. The participants walked on a 15 m straight walk way at their self-selected gait speed. The participants who use a cane for regular gait training used a cane in five gait conditions during the measurements of this study. The order of gait conditions began with the no-AFO condition, followed by the four AFO stiffness conditions implemented in a random order using simple randomization. The randomization was performed by computer-generated random numbers. The participants were allowed to practice sufficient walking under each condition. They were allowed to sit and rest at any time during the experiment. We also selected the best improvement condition (best condition) from the five-gait conditions for each participant and compared the best condition with the other conditions because the stiffness of the AFO should be tuned based on the body function and gait performance of each user in a clinical setting. Previous studies have reported that the AFO and AFO stiffness can affect various gait parameters, particularly the gait speed [4], [6], [23]. Furthermore, gait speed is related not only to daily activities, but also to the participation in society [24], [25]. Therefore, in this study, the greatest improvement in the gait speed in the five-gait conditions was defined as the best condition.

### C. Data Analysis and Statistical Analysis

The 2D coordinates of the body keypoints in each frame estimated by OpenPose were used to calculate the spatiotemporal gait parameters. The missing keypoint data were interpolated using the third-order spline method. Because pose estimation by a single RGB camera sometimes switched keypoints between the left and right lower limbs, the switch was identified and corrected based on the keypoint coordinates of the left and right heels [19]. The keypoint data were filtered with a 6 Hz cut-off frequency fourth-order low-pass Butterworth filter. The gait speed was calculated from the average speed of the mid-hip keypoint in the direction of the straight walk [19]. The timing of the initial contact and toe-off was defined based on the mid-hip, knees, ankles, and big toe keypoints, referring to previous studies, [26], [27] because individuals with stroke have a large variation in initial contact. The stance time is defined as the period from the observed side initial contact to the observed side toe-off, whereas the swing time is defined as the period from the observed side toe-off to the observed side initial contact. Because the participants had hemiplegia, cadence (steps/min) was calculated from the elapsed time between the two steps. The accuracy of the spatiotemporal gait parameters using OpenPose has already been investigated under various gait conditions, and it maintains good accuracy under these gait conditions [28], [29].

To compare the gait speed, cadence, step length, stance phase, and swing phase among the six conditions, including the best condition, a one-way repeated measures analysis of variance (repeated ANOVA) was used in this study. The Friedman’s test was used for nonparametric data. To address issues of data independence and bias as much as possible, the Wilcoxon signed-rank test with correction by the Holm method was performed as a post hoc test for all comparison of this study. Statistical analyses were performed using the R version 3.6.3 software (CRAN, freeware). The Effect size of the post hoc test was also calculated using *r* value, and *r* was defined as small (<0.3), medium (0.3 – 0.5), and large (>0.5). The statistical significance was set to $p < 0.05$.

## III. Results

**FIGURE 3.**
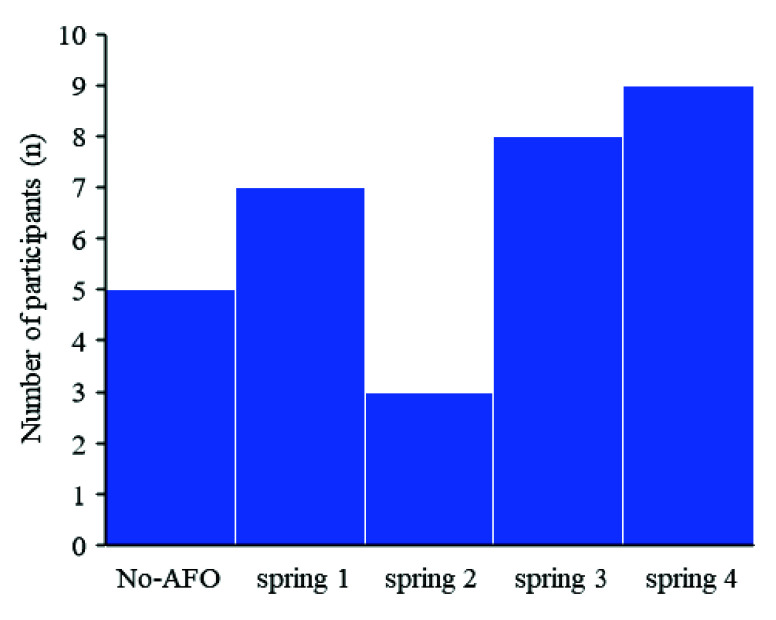
Number of the best gait speed improvement condition (the best condition) under each condition.

Fig. 3 shows the number of best gait speed improvements for each condition. In many cases in this study, the gait speed increased the most when the participants walked with an AFO with a type of spring.

**FIGURE 4.**
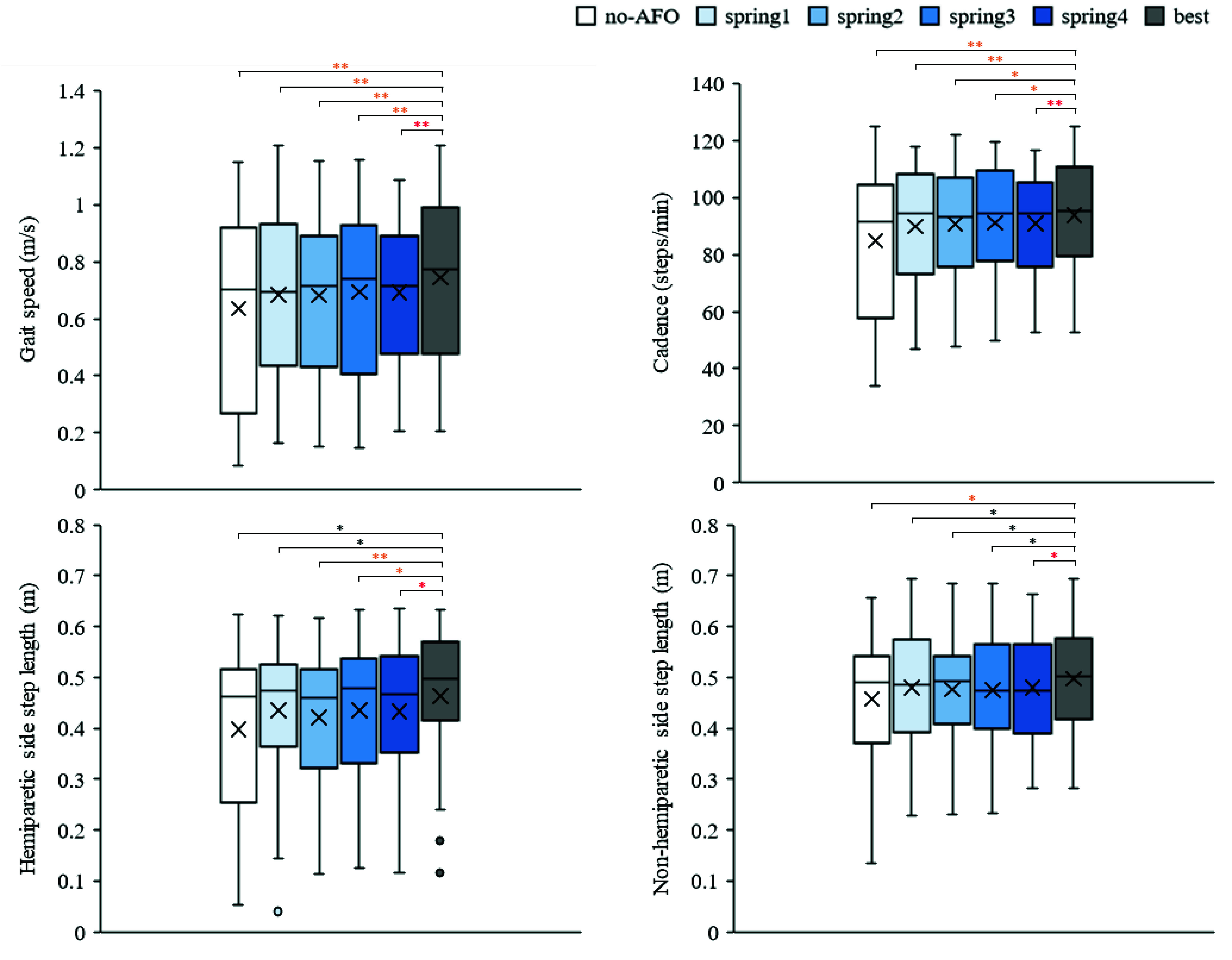
Results of gait speed, cadence, and step length on each condition. Boxes and horizontal lines represent ranges of Q1, Q3, and median values. $\times $ and circles indicate mean and outlier, respectively. ${}^{\ast }$ and ${}^{\ast \ast }$ indicate significant difference ( ${p} < 0.05$ and ${p} < 0.01$, respectively). Color of the ${}^{\ast }$ indicates the effect size r, which is black: small (<0.3), orange: medium (0.3 - 0.5), and red: large (>0.5) respectively.

Fig. 4 shows the gait speed, cadence, and step length on both the sides under the six conditions. These gait parameters differed significantly between the six conditions (gait speed, $p < 0.001$ [repeated ANOVA]; cadence, $p < 0.001$ [repeated ANOVA]; hemiparetic side step length, $p~< 0.001$ [Friedman’s test]; and non-hemiparetic side step length, $p=0.009$ [repeated ANOVA]). The best conditions for gait speed, cadence, and step length on both the sides significantly increased compared with those of the other conditions. The mean difference of the gait speed, cadence, hemiparetic side step length, and non-hemiparetic side step length between the best and the no-AFO conditions were 0.11 m/s, 8.79 steps/min, 0.06 m, and 0.04 m, respectively.

**FIGURE 5.**
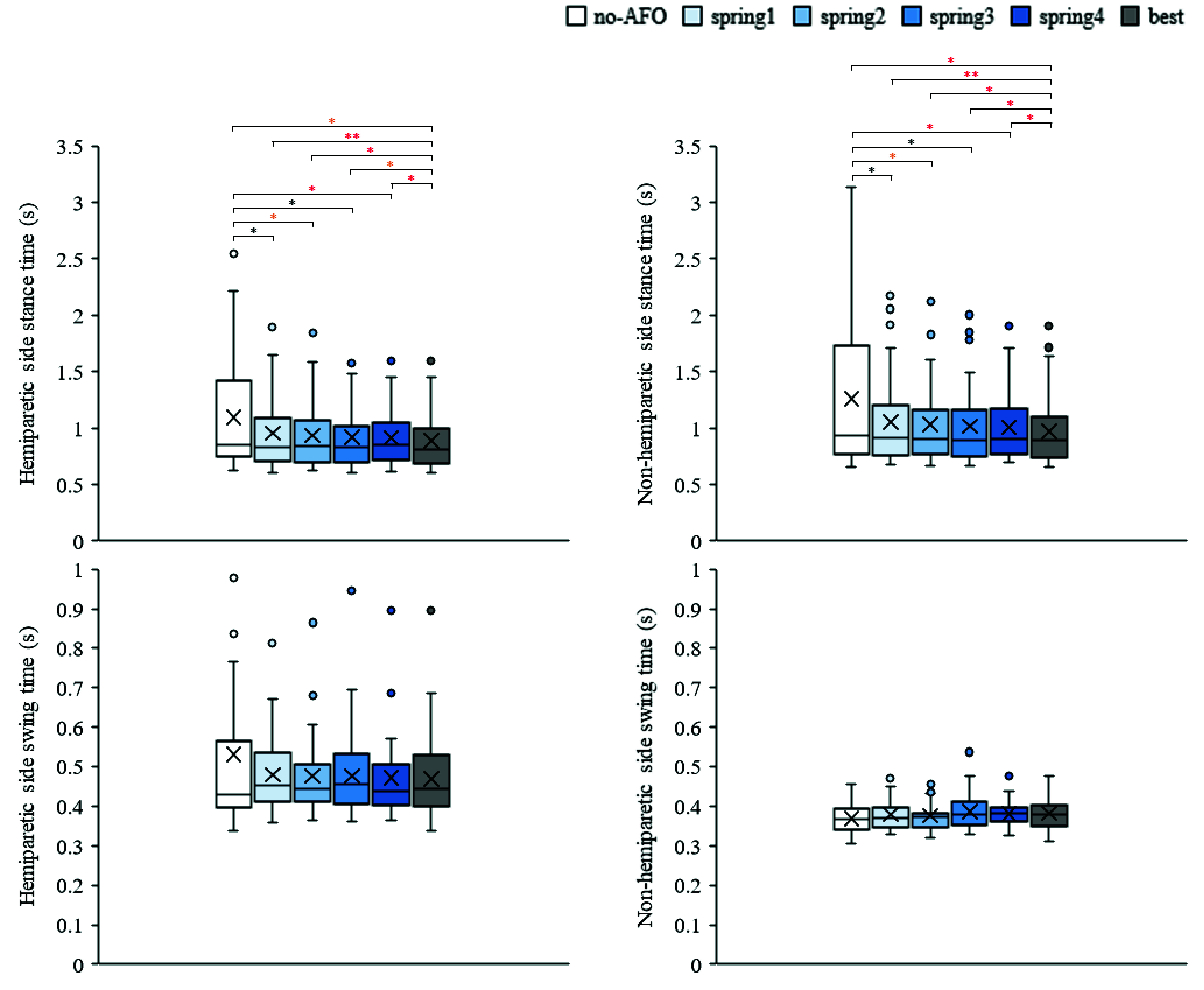
Results of stance time and swing time on each condition. Boxes and horizontal lines represent ranges of Q1, Q3 and median values. $\times $ and circles indicates mean and outlier, respectively. ${}^{\ast }$ and ${}^{\ast \ast }$ indicates significant difference ( ${p} < 0.05$ and ${p} < 0.01$, respectively). Color of the ${}^{\ast }$ indicates the effect size r, which is black: small (<0.3), orange: medium (0.3 - 0.5), and red: large (>0.5) respectively.

Fig. 5 shows the stance time and swing time of the hemiparetic and non-hemiparetic sides for the six conditions. The stance time of both the sides differed significantly among the six conditions (hemiparetic side stance time, $p < 0.001$ [Friedman test]; non-hemiparetic side stance time, $p < 0.001$ [Friedman test]). Under the best condition, the stance time of both the sides significantly decreased compared to those of the other conditions. Moreover, there were significant differences in the stance time between the no-AFO condition and all the spring conditions on both the sides. There were no significant differences in the swing time on either side among the six conditions (hemiparetic side swing time, $p =0.597$ [Friedman’s test]; non-hemiparetic side stance time, $p=0.505$ [Friedman’s test]). The mean difference of the hemiparetic side stance time, non-hemiparetic side stance time, hemiparetic side swing time, and non-hemiparetic side swing time were -0.21 s, -0.29 s, -0.06 s, and 0.01 s, respectively.

## IV. Discussion

This study aimed to verify the effect of AFO stiffness adjustment on the gait ability of the individuals with stroke using the markerless gait analysis system. The spatiotemporal parameters in individuals with stroke were measured under various conditions of AFO stiffness using a single RGB camera and human pose estimation. Furthermore, the best gait speed improvement condition was set from the five-gait conditions for each participant and the best condition was compared with other conditions assuming a clinical setting. The results showed that the best condition significantly improved some spatiotemporal parameters compared with the other conditions.

In the best condition, the gait speed significantly increased compared to the other conditions. The immediate mean differences in the gait speed between the best and other conditions ranged from 0.05 to 0.11 m/s. A previous study reported a minimally clinically important difference in gait speed in individuals with stroke who underwent a stroke rehabilitation trial over three weeks, which ranged from 0.06 to 0.14 m/s [30]. Although the results of this study showed immediate effects, the magnitude of improvement of this study was similar to the range of the minimally clinically important difference during the three weeks rehabilitation [30]. Therefore, this effect of AFO stiffness adjustment based on the gait speed of the participants seems to be sufficient as an immediate treatment effect. Furthermore, the cadence and step length of both the sides significantly increased in the best condition compared to the other conditions. These parameters were lower in stroke patients than in age-matched healthy adults [2]. The step length and cadence are also important spatiotemporal parameters related to fall risk and gait ability [31], [32]. These parameters are also presumed to improve concomitantly with optimal stiffness adjustments based on the gait speed.

Although there were no statistically significant differences in the swing time of both the sides during gait, the stance time of both the sides in all the spring conditions significantly decreased compared with the no-AFO condition. Moreover, the stance time of both the sides in the best condition significantly decreased compared to that in the other conditions. The duration of the stance phase, especially the double-stance phase, was higher in patients with stroke than in healthy adults [33]. An AFO with a plantarflexion resistance function can improve the heel and ankle rocker function during gait. An AFO with a plantarflexion resistance function could assist individuals with stroke in shifting their weight to their hemiparetic lower limb [6], [7]. As the gait speed and step length improved, the stance time of both the sides seemingly decreased because of smooth weight acceptance and rhythmic gait.

This study has a few limitations that should be addressed. First, this study did not measure kinematic and kinetic outcomes such as joint angles and joint moments, and only immediate effect were investigated. Although the spatiotemporal gait parameter is one of the primary outcomes of post-stroke gait function, important findings may be obtained by measuring joint kinematics. Pose estimation using a single RGB camera has great potential, as it could simultaneously calculate other related spatiotemporal parameters and joint angles if the accuracy issues are resolved. Also, an IMU and triangulation using a multicamera can be used to analyze the joint angles. Second, this study did not focus on the dorsiflexion resistance function of the AFO. This function can also contribute to the gait performance individuals with stroke. Further studies should investigate the effect of on plantarflexion and dorsiflexion stiffness during gait in individuals with stroke patients. Third, the best condition for AFO stiffness was limited to the gait speed. Therapists use several treatment strategies and make decisions based on the treatment goals and gait dysfunction of each patient. Sometimes, they focus on improving the gait speed, whereas sometimes the focus is on different gait parameters. This study defined the greatest improvement in gait speed from these gait conditions as the best condition, because gait speed is one of the most important parameters in stroke gait rehabilitation. Future studies should investigate the indicators considered important by therapists and compare the AFO stiffness selected by therapists with other conditions. Despite these limitations, the findings of this study suggest the effectiveness of AFO stiffness adjustment based on the gait ability of the user and the usefulness of a markerless gait analysis method using a single RGB camera.

## V. Conclusion

The purpose of this study was to investigate the effect of AFO stiffness adjustment based on the gait ability of the user using a markerless gait analysis system. To easily measure the spatiotemporal gait parameters in individuals with stroke in a clinical setting, we used a single RGB camera and a pose estimation system. From the results of this gait analysis method, the AFO stiffness adjustment based on gait speed significantly improved compared to the other conditions. The proposed method may also have the potential for motion analysis in various patients.

## Conflicts of Interest

The authors have no conflicts of interest in relation to the work reported here.
